# Public health round-up

**DOI:** 10.2471/BLT.17.010317

**Published:** 2017-03-01

**WHO staff walk the talk**

Since its launch in January 2016, the World Health Organization’s (WHO) Walk the Talk initiative has gone global, with staff members at Headquarters, Regional and Country offices joining the movement to keep fit and healthy at work. These staff members are from the Jordan Country Office.

**Figure:**
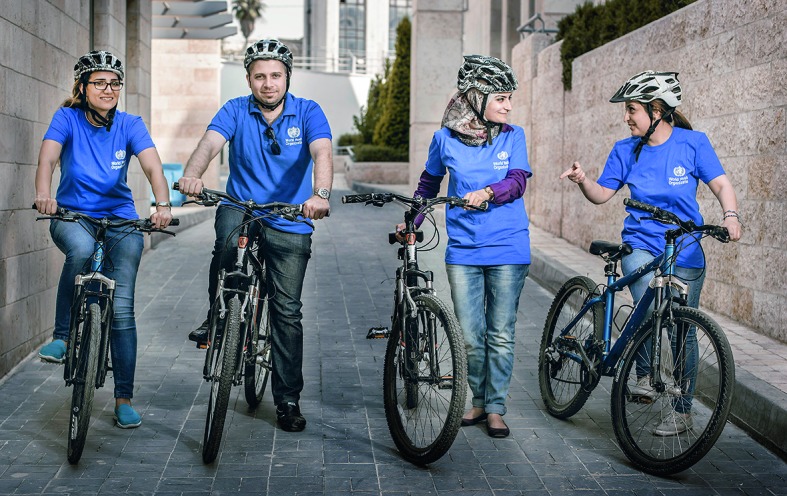
WHO Jordan

## WHO Director-General election

The WHO Executive Board has selected three candidates to be presented to the World Health Assembly as nominees for the post of Director-General.

The three nominees were announced at a public meeting on 25 January. They are Dr Tedros Adhanom Ghebreyesus, nominated by the Government of Ethiopia, Dr David Nabarro, nominated by the Government of Great Britain and Northern Ireland, and Dr Sania Nishtar, nominated by the Government of Pakistan.

WHO’s Member States will select one of the three nominees by vote at the World Health Assembly in May. The new Director-General will take office 1 July 2017.

http://www.who.int/dg/election/

## Early cancer diagnosis

New guidance from WHO, launched ahead of World Cancer Day on 4 February, aims to improve the chances of survival for people living with cancer by ensuring that health services can focus on diagnosing and treating the disease earlier.

New WHO figures released recently indicate that each year 8.8 million people die from cancer, mostly in low- and middle-income countries.

Even in countries with optimal health systems and services, many cancer cases are diagnosed at an advanced stage, when they are harder to treat successfully.

"Diagnosing cancer in late stages, and the inability to provide treatment, condemns many people to unnecessary suffering and early death," said Dr Etienne Krug, Director of WHO’s Department for the Management of Noncommunicable Diseases, Disability, Violence and Injury Prevention.

"By taking the steps to implement WHO’s new guidance, health-care planners can improve early diagnosis of cancer and ensure prompt treatment, especially for breast, cervical and colorectal cancers. This will result in more people surviving cancer. It will also be less expensive to treat and cure cancer patients."

All countries can take steps to improve early diagnosis of cancer, according to WHO’s new *Guide to cancer early diagnosis*.

The three steps to early diagnosis are: improving public awareness of different cancer symptoms and encouraging people to seek care when these arise; investment in strengthening and equipping health services and training health workers; and ensuring people living with cancer can access safe and effective treatment, including pain relief, without incurring personal or financial hardship.

http://www.who.int/cancer/publications/cancer_early_diagnosis

## Measles and rubella vaccination in India

India has launched one of the world’s largest vaccination campaigns against measles and rubella.

The campaign started on 7 February to vaccinate more than 35 million children in the age group of nine months to 15 years.

The first phase of the campaign was expected to accelerate the country’s efforts to eliminate measles which affects an estimated 2.5 million children every year, killing nearly 49 000 of them.

The campaign also marks the introduction of rubella vaccine in India’s childhood immunization programme to address congenital rubella syndrome which causes birth defects such as irreversible deafness and blindness in nearly 40 000 children every year.

India has made important efforts and gains against measles in recent years. Measles deaths have declined by 51% from an estimated 100 000 in the year 2000 to 49 000 in 2015.

This has been made possible by significantly increasing the reach of the first dose of measles vaccine, given at the age of nine months under routine immunization, from 56% in 2000 to 87% in 2015.

In 2010, India introduced the second dose of measles-containing vaccine in its routine immunization programme to close the immunity gap and accelerate measles elimination.

Nearly 118 million children aged nine months to 10 years were vaccinated during mass measles vaccination campaigns between 2010 and 2013 in some Indian states.

Last month’s campaign was the first in the series to cover a total of 410 million children across the country over the next two years.

“Apart from improving the life-chances of millions of children in India, the campaign is expected to have a substantial effect on global measles mortality and rubella control targets as India accounts for 37% of global deaths from measles,” said Dr Poonam Khetrapal Singh, WHO Regional Director for South-East Asia.

http://www.searo.who.int/mediacentre/features/2017/india-measles-rubella-vaccination-campaign

## Health needs soar in eastern Ukraine

A large shipment of WHO interagency emergency health kits was sent to the eastern part of Ukraine to help meet immediate health needs in response to the recent intensification of fighting and shelling there.

Thousands of civilians are living in sub-zero temperatures and many others are injured and urgently in need of life-saving medical care.

“The humanitarian situation is dire in the midst of winter as electricity, heat, water and basic services have been disrupted in large parts of Donetsk,” said Dr Marthe Everard, WHO Representative in Ukraine. “Our efforts are focused on ensuring civilians are protected from hostilities and have access to basic services, including essential health care.”

The Government of Ukraine has organized voluntary medical evacuations for children and vulnerable residents of Avdiivka, a city north of Donetsk, but thousands more in the area may need to be evacuated in the coming weeks due to continued shelling and extreme cold.

WHO is working with the Government of Ukraine and health partners to ensure, and coordinate, timely delivery of the emergency health kits to hospitals and health clinics. Material for preventive services such as immunization and the detection and treatment of common diseases will also be provided – in government-controlled areas, non-government-controlled areas and buffer zones.

Together with other United Nations agencies and health partners, WHO is making efforts to reduce people’s exposure to the cold and to ensure that they have access to heated shelters, regular hot meals and warm clothing.

http://www.euro.who.int/en/health-topics/emergencies/pages/news/news/2017/02/health-needs-soar-as-fighting-flares-in-eastern-ukraine

**Cover photo**

This man and his wife were living with their children in a settlement for displaced indigenous Emebera Chami people in Caqueta, Colombia in 2013. The United Nations High Commissioner for Refugees (UNHCR) worked with local organization Yapawayrya to set up a settlement for 35 families displaced by armed guerrillas in 2005 (© UNHCR/ Sebastian Rich).

**Figure:**
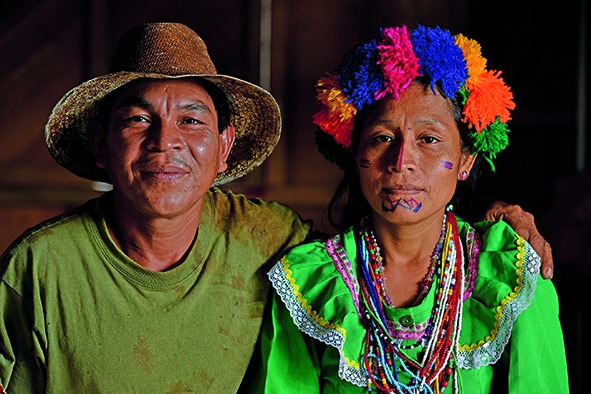
© UNHCR/Johan Bävman

## Trauma care in Iraq

The Government of France, through the European Union Civil Protection Mechanism, has swiftly responded to an appeal by WHO and the Iraqi Ministry of Health, for medicines and medical supplies to manage the overwhelming number of injured people coming from east Mosul, WHO said on 30 January.

The shipment consists of 20 surgical kits sufficient to conduct 2000 surgical procedures, half of which will go to each of the two main referral hospitals in Erbil, northern Iraq. It also contains life-saving medicines sufficient to serve the needs of 12 000 patients.

So far more than 3300 injured people from east Mosul, including many women and children, have been treated in the two hospitals since 17 October 2016.

Since the beginning of the Mosul operations, the Federal Ministry of Health and the Regional Ministry of Health, Kurdistan Regional Government, with the support of WHO, have provided additional training for 60 doctors.

WHO has also provided medical supplies for patients in and around camps, and in hospitals within Mosul and other parts of Iraq. 

As the frontlines move towards west Mosul, WHO and partners anticipate a higher number of trauma cases in the coming months.

http://www.emro.who.int/irq/iraq-news/who-scales-up-response-to-critical-trauma-needs-mosul.html

## Rehabilitation 2030: a call for action

A meeting was held at WHO headquarters in Geneva on 6–7 February to address the need for rehabilitation particularly in low- and middle-income countries.

Accessible and affordable rehabilitation is necessary for many people, enabling them to remain as independent as possible, to participate in education, to be economically productive, and fulfil meaningful life roles.

Bringing stakeholders together provided a valuable opportunity to discuss coordinated action to raise the profile of rehabilitation as a health strategy relevant to the whole population, across the lifespan and across the continuum of care.**

http://www.who.int/disabilities/care/rehab-2030

**Looking ahead**

**7 April – World Health Day**: this year’s theme is “depression, let’s talk”

**22–31 May – 70th World Health Assembly**, Geneva, Switzerland

**31 May – World No Tobacco Day**: this year’s theme is “tobacco: a threat to development”

